# New-Onset Rheumatoid Arthritis in Pregnancy: A Case Report

**DOI:** 10.7759/cureus.64899

**Published:** 2024-07-19

**Authors:** Aiysha Gul, Muzamil Khan, Satkarjeet Kaur Gill, Rizwanullah, Karthiga Vasudevan, Kiya Gurmessa, Sophia Tahir

**Affiliations:** 1 Obstetrics and Gynecology, Mardan Medical Complex, Mardan, PAK; 2 Internal Medicine, The George Washington University School of Medicine and Health Sciences, Washington DC, USA; 3 Internal Medicine, Jagare Ridge Medical Clinic, Edmonton, CAN; 4 Internal Medicine, Hayatabad Medical Complex, Peshawar, PAK; 5 Preventive Medicine, Yerevan State Medical University, Yerevan, ARM; 6 Public Health, Johns Hopkins University, Baltimore, USA; 7 Internal Medicine, Windsor University School of Medicine, Cayon, KNA

**Keywords:** female, immunological changes, autoimmune disease, pregnancy, rheumatoid arthritis

## Abstract

Rheumatoid arthritis (RA) is extremely uncommon during pregnancy. The alterations in the immune system that occur to support the developing fetus make the onset of RA during this period unlikely. In this case report, we describe a 26-year-old pregnant woman who presented with bilateral symmetrical pain in her hands, wrists, and ankles at 24 weeks of gestation. After a thorough evaluation, she was diagnosed with active RA based on clinical symptoms and laboratory findings, including elevated inflammatory markers, positive RA factor, and anti-cyclic citrullinated peptide antibodies. Treatment was initiated with hydroxychloroquine (HCQ), prednisolone, and paracetamol, resulting in significant symptom improvement and no postpartum complications. The patient gave birth to a healthy baby via vaginal delivery, highlighting the management challenges and outcomes linked to RA during pregnancy.

## Introduction

Rheumatoid arthritis (RA) is a chronic systemic autoimmune disorder that impacts women three times more often than men, typically during their most productive and childbearing years [1]. The progression of RA frequently alters during pregnancy, with around 50% of pregnant women experiencing low disease activity. Between 20% and 40% of these women achieve remission by the third trimester; however, nearly 20% will experience a worsening or moderate-to-high disease activity, necessitating additional therapeutic intervention [2].

The remission of RA during pregnancy can be linked to the shift from Th1-mediated immunity to Th2. This shift suppresses Th1 cells while enhancing Th2 cells, which promotes humoral and antibody-based immunity. RA is a chronic inflammatory condition mainly driven by immunological dysfunction and T-cell infiltration. These T cells release cytokines, causing inflammation and arthritis due to cartilage destruction and systemic symptoms. The primary characteristic of RA is inflammatory synovitis, usually affecting the peripheral joints [3].

The postpartum period is a time when women are especially susceptible to RA flares and the initial onset of RA. There is a higher incidence of RA following the first pregnancy, particularly within the first nine months. Up to 90% of patients with RA may experience postpartum flares, typically within the first three months, and these flares are more common after the first pregnancy [4].

Due to the immunological changes that occur during pregnancy, autoimmune diseases are rare during this period. There are only a few documented cases of RA in pregnant women. In this report, we present the case of a 26-year-old woman who, at 24 weeks of gestation, experienced bilateral symmetrical pain in her hands, wrists, and ankle joints. She was subsequently diagnosed with active RA.

## Case presentation

A 26-year-old primigravida, who was 24 weeks pregnant, visited the outpatient department of Hayatabad Medical Complex in Peshawar. She had been experiencing bilateral symmetrical pain in her hands, wrists, and ankles for the past three months. She reported no previous comorbidities and no family history of autoimmune diseases. Upon further questioning, she mentioned experiencing morning stiffness in the affected joints for about 30 minutes, which improved with physical activity.

During the rheumatological examination, she exhibited tenderness in eight proximal interphalangeal joints, as well as in her wrist and ankle joints. Her range of motion was restricted due to pain. The rest of her abdominal, respiratory, neurological, cardiovascular, and dermatological examinations were unremarkable. 

Our differential diagnosis included RA, systemic lupus erythematosus, carpal tunnel syndrome, and hypothyroidism. Hypothyroidism was ruled out as the patient exhibited no significant features of this condition, and her thyroid profile was normal. Carpal tunnel syndrome was also excluded due to negative results for Tinel's Sign and Phalen's Test, along with normal nerve conduction studies (NCS) and electromyography (EMG) studies. She presented no clinical features of SLE other than rheumatological signs, and her ANA and dsDNA tests were negative. Although RA is rare during pregnancy, some cases have been reported. Considering this possibility, we investigated her for RA, which was confirmed by the positivity of rheumatoid factor (RF) and anti-CCP antibodies.

Laboratory investigations revealed leukocytosis, elevated inflammatory markers, and significantly positive RA and anti-cyclic citrullinated peptide antibodies (anti-CCP). Other tests, including anti-neutrophilic antibodies (ANA), extractable nuclear antigen (ENA), and anti-double-stranded DNA (anti-dDNA), were negative (Table 1). Her Disease Activity Score 28 (DAS 28) was calculated at 4.3, indicating moderate severity.

**Table 1:** Laboratory investigations. WBC, white blood cell count; mcL, microliter; mg/dL, milligram per deciliter; IU/mL, international units per milliliter; CRP, C-reactive protein; ESR, erythrocyte sedimentation rate; RF, rheumatoid factor; anti-CCP, anti-cyclic citrullinated peptide

Labs	Reference range	Day 1	Day 3	After one month
WBCs (x10^3^/mcL)	4-11	13	12.5	7.4
Hemoglobin (mg/dL)	11.5-17.5	13.5	12.9	12.4
Platelets counts (x10^3^/mcL)	150-450	268	261	247
CRP (mg/dL)	<0.5	5.2	3.8	0.8
ESR (mm/1st hour)	0-20	47	41	27
RF (IU/mL)	<20	364	N/A	N/A
Anti-CCP (IU/mL)	<20	789	N/A	N/A

**Figure 1:**
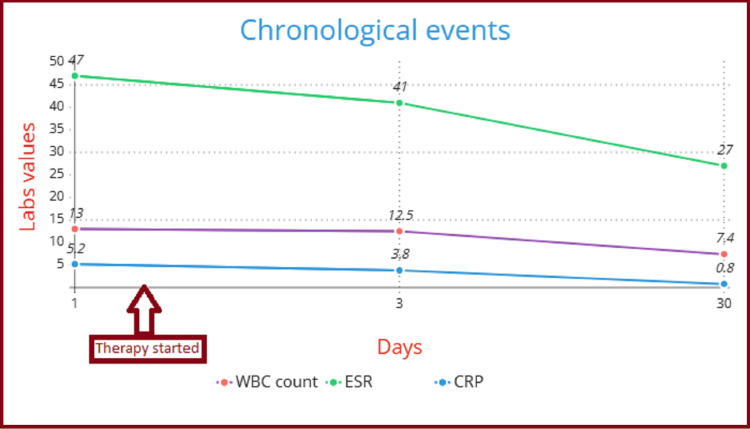
Trends in laboratory investigations from admission to follow-up. ESR, erythrocyte sedimentation rate; CRP, C-reactive protein

Considering this a case of active RA, she was started on oral hydroxychloroquine (HCQ) 400 mg per day, oral prednisolone 10 mg per day, and analgesic paracetamol. After a one-month follow-up, her clinical condition had improved, along with a reduction in inflammatory markers (Figure 1). At 38 weeks of gestation, she had a normal vaginal delivery, with a baby weighing 3.5 kg. No anomalies were detected during the pediatric assessment.

## Discussion

The precise mechanism through which RA tends to improve during pregnancy remains incompletely understood. However, it is thought that pregnancy creates a condition of immune tolerance because of the presence of the semi-allogeneic fetus. This tolerance encompasses several immunological mechanisms, such as thymus regression, decreased natural killer cell function, and a shift in immune reaction from Th1 cells toward Th2 dominance [5]. Additionally, molecules expressed by the syncytiotrophoblast, such as decay-accelerating factor and membrane cofactor protein, inhibit complement activation, protecting embryonic cells from complement-mediated damage [6]. From a cellular immunity standpoint, the activation of T cells at the maternal-fetal interface is suppressed by the local production of indoleamine 2,3-dioxygenase, an enzyme that breaks down tryptophan-an amino acid crucial for T-cell activation [7]. There have been a total of five documented cases of RA occurring during pregnancy [8-11].

Non-steroidal anti-inflammatory drugs (NSAIDs) are generally considered safe for use during pregnancy; however, they are not recommended during the final trimester due to the potential risk of premature closure of the ductus arteriosus. If symptoms arise or worsen during pregnancy, NSAIDs are the preferred treatment option. Glucocorticoids, such as prednisone, are commonly prescribed when NSAIDs are not advisable. The aim is to administer prednisone at the lowest effective dose, usually not exceeding 10 mg daily, to manage the condition. Nonfluorinated glucocorticoids like prednisolone, prednisone, and methylprednisolone pass through the placenta in small amounts and are metabolized into inactive forms before reaching the fetus. Hence, they are generally regarded as safe for use during pregnancy at low to moderate doses [12].

Disease-modifying antirheumatic drugs (DMARDs) such as HCQ, sulfasalazine (SSZ), and azathioprine (AZA) can be continued for patients who do not respond adequately to NSAIDs or prednisone. For moderately active disease, HCQ and/or SSZ may be prescribed. Methotrexate (MTX) and leflunomide (LEF) should be avoided during pregnancy [13]. Biological treatments such as tumor necrosis factor (TNF) inhibitors may be maintained during pregnancy based on the particular medication and a careful assessment of the individual risks and benefits involved [14].

We initiated treatment for our patient with HCQ at a daily dose of 400 mg, oral prednisolone at 10 mg per day, and paracetamol for pain relief. After one month of monitoring, the patient experienced notable symptom improvement, with no complications or disease flare-ups postpartum. The baby was delivered vaginally without complications, weighed within normal limits, and showed no abnormalities.

While many women with RA often experience improvement during pregnancy, it is crucial not to overlook disease flares or mistake them for pregnancy-related symptoms. Patients presenting with both typical and atypical RA symptoms should undergo a thorough evaluation for various rheumatologic conditions to ensure accurate diagnosis and timely initiation of suitable treatment. The initial onset of RA during pregnancy could indicate a potentially more severe clinical trajectory. Further research focusing on this distinct patient population is essential to better understand the long-term prognosis of their condition.

## Conclusions

This case report underscores the complexities of managing RA during pregnancy, where disease progression varies widely among individuals. Pregnancy-induced immunological changes often result in improved RA symptoms, influenced by shifts in immune responses and hormonal dynamics. However, some patients may experience disease flares or new-onset RA during pregnancy, necessitating vigilant monitoring and tailored therapeutic approaches. Our patient, diagnosed with active RA at 24 weeks of gestation, responded positively to treatment with HCQ, prednisolone, and paracetamol, achieving significant symptom relief and favorable postpartum outcomes. This highlights the critical role of early diagnosis and appropriate management in mitigating potential complications and ensuring the well-being of both mother and fetus. Further research is essential to better understand the underlying mechanisms of RA's behavior during pregnancy and optimize therapeutic strategies for pregnant women with RA.
